# Characteristics, crack use, housing situation and psychosocial problems of people in the open drug scene in cologne, Germany – results of a cross-sectional survey

**DOI:** 10.1186/s12954-025-01256-2

**Published:** 2025-06-05

**Authors:** Daniel Deimel, Lucie Feldmann, Norbert Scherbaum, Christine Firk, Simon Fleißner

**Affiliations:** 1 Faculty of Social Science, Nuremberg University of Applied Sciences, Nuremberg, Germany; 2 Department of Psychiatry and Psychotherapy, Medical Faculty, LVR University Hospital Essen, University of Duisburg-Essen, Essen, Germany; 3 Health Department, City of Cologne, Cologne, Germany; 4 Catholic University of Applied Sciences North Rhine Westphalia, Aachen, Germany; 5 Institut of Addiction Research Frankfurt, Frankfurt University of Applied Sciences, Frankfurt, Germany

**Keywords:** Open drug scene, Harm reduction, Substance use, Crack, Heroin, Homelessness

## Abstract

**Background:**

Open drug scenes are characterized by public gatherings of people who use drugs (PWUD) for the purpose of drug consumption and trade, often related to poor health conditions as risk of overdose, a higher prevalence of infectious disease and social exclusion. This cross-sectional survey investigated the characteristics, substance use patterns, and support needs of PWUD within the open drug scene at Neumarkt in Cologne (ODSC) in Germany.

**Methods:**

A cross-sectional survey was conducted between April and June 2023 among PWUD frequenting the ODSC. A structured questionnaire, comprising sociodemographic information, substance use patterns, overdose history, and psychosocial support needs was used.

**Results:**

A total of 119 participants were surveyed, with a majority identifying as male (79%) and an average age of 42 years. Heroin was the most frequently consumed substance (64.7%), followed by alcohol (56.3%) and crack/cocaine (21%). People who use crack (PWUC) were younger (mean age 36.6) and more likely to be homeless (56%) than respondents who are not using crack. The most mentioned support needs included housing (69.5%), bureaucratic assistance (60.2%), and health related support (51.7%).

**Conclusion:**

The ODSC presents a complex risk environment where homelessness, limited access to healthcare due to lack of health insurance, and frequent substance use exacerbate health and social challenges. Expanding harm reduction services, including housing first initiatives, and low-threshold opioid substitution treatment access for individuals without health insurance, is crucial to address these issues.

## Introduction

An open drug scene (ODSC) is usually understood as a gathering or network of people who trade and use drugs in public spaces [1, 2]. ODSC are mostly limited to a specific geographic area [3] and for some people who use drugs (PWUD) the ODSC is their central living space [2]. Waal and colleagues summarize: “When drug users congregate to sell and use drugs in public spaces, this phenomenon is referred to as an open drug scene” [4]. However, it remains difficult to establish a universal definition for the varying phenomenon of an ODSC. Bless and colleagues describe three types of drug scenes (concentrated open scenes, dispersed open scenes, hidden scenes) and illustrate that ODSC can vary significantly [5]. Applying the “risk environment” concept to harm reduction, as proposed by Rhodes: “We can define the risk environment as the space - whether social or physical - where various factors interact to increase the chances of drug-related harm” [6], it reveals the harms associated with an ODSC. Rhodes thus focuses not on changing the behavior of PWUD of an ODSC but rather on changing the environment to improve the health of PWUD [6]. ODSC can be perceived as a burden by residents and raising public policy issues [7, 8]. Therefore, ODSC can provoke a discussion who is allowed to use public space and often PWUD are marginalized and problematized if they are visible in the neighborhood [9, 10]. However, they are especially a place that brings various health and personal problems and burdens for the people in the ODSC [4, 11, 12]. Research clearly shows that PWUD intravenous have poor general health [11, 13, 14]. Infectious diseases are significantly more prevalent compared to the general population [13]. In addition, PWUD in ODSC are exposed to higher psychological stress than PWUD in opioid substitution therapy (OST) and in general mental illnesses are also significantly more common [15]. All in all the mortality of PWUD is higher than that of the general population [16], making the group of PWUD encountered in ODSC undoubtedly a heavily burdened group in terms of health [11, 14].

## Open drug scenes in Germany

The most common used illicit drug in ODSC in Germany is heroin [11, 12]. In recent years, an increase of the use of crack in ODSC in Germany has been reported [11, 17]. Crystallized cocaine is called crack and is smoked, producing a faster but shorter and more intense high. While crack use had previously been observed mainly in Frankfurt a. M., Hamburg, and Hanover [18] the reported increase in other cities of Germany is particularly relevant as it can change the dynamics of ODSC, and the services provided by harm reduction facilities need to adapt accordingly [19]. Since 2018, both an increase in cocaine use and inhalation of crack and heroin in general has been reported in consumption rooms in the federal state of North Rhine-Westphalia, where Cologne is located [20]. Reports of drug aid facilities from 2021 to 2022 show that crack use has increased, especially at first in northern and western parts of Germany and since 2022 also in southern Germany [17, 21], and therefore this contribution has a special focus on crack use and the motives of crack use. This study presents the living conditions and characteristics of PWUD in Cologne’s largest ODSC, located at the central square “Neumarkt”. Cologne, with 1.08 million inhabitants, is the fourth-largest city in Germany after Berlin, Hamburg, and Munich [22]. According to personal communication from the Drug and Addiction Commissioner of the City of Cologne, approximately 2,000 individuals are currently receiving OST. Given that approximately 50% of people who are using heroin are in OST in Germany, we estimate that 4,000 people who are using heroin are currently living in Cologne [23]. Additionally, not everyone on the ODSC is using heroin. Currently, there are two drug consumption rooms in Cologne. There are plans of opening a third [24]. Managing drug use and the phenomena of ODSC is a complex undertaking that requires coordinated collaboration between politics, police, justice, and social work [25] and therefore need insights into the local ODSC. This cross-sectional survey investigated the characteristics, substance use patterns, and support needs of PWUD within the ODSC at Neumarkt in Cologne in Germany. This study is the first of its kind to survey the ODSC in Cologne.

## Method

Participants and Data Collection.

A total of 119 participants took part in the survey, which was conducted between April 29, 2023, and June 27, 2023, in Cologne. The participants were individuals who identified as part of the ODSC at Cologne’s Neumarkt, were 18 years of age or older and were able to speak German or English. If the participants were able to be interviewed, intoxication was not a criterion for exclusion. The contact with the PWUD was facilitated by street workers from the Cologne Health Department. PWUD were actively approached by an interviewer and informed about the study. 10 € supermarket vouchers were offered as incentives to encourage participation. Individuals willing to participate were interviewed face-to-face using a standardized questionnaire with 21 closed and open questions. The questionnaire was completed by the interviewer during the in-person interview. The duration of the interviews was between 20 and 40 min. The survey was administered in both German and English.

### Measures

The questionnaire included the following topics:

- Sociodemographics (age, gender, migration background, income, housing situation).
- Substance use (24-hour prevalence, mode of heroin and cocaine administration, time of first and last cocaine, crack, and heroin use, as well as the average number of consumption events for cocaine, crack, and heroin per day, number of drug overdoses).
- Motives for crack use were assessed with open questions: “Why did you smoke cocaine (crack)?” and “How do you feel when you smoke cocaine (crack)?”
- Health insurance status.
- Use of support services (opioid substitution therapy, drug consumption rooms).
- (Psychosocial) support needs (e.g. bureaucratic matters like getting an ID card, health insurance or housing).
- System-related recommendations and individual wishes of the respondents.

### Statistical analysis

Data analysis was conducted using IBM SPSS Statistics 25.0. Chi-square tests were used for group comparisons of categorical variables. *P*-values of < 0.05 were considered statistically significant. Answers to open questions were translated from German to English.

### Ethical considerations

Participants were fully informed about the study, including its purpose, the voluntary nature of participation, and their right to withdraw at any time without disadvantage. Verbal informed consent was obtained and documented by the interviewer. No personal identifiers were collected, and all data were gathered anonymously. The study was approved by the Ethics Committee of the Catholic University of Applied Sciences Aachen (AZ2023-02). Although the study was not registered in a public trial database, it was conducted in line with fundamental ethical principles of the Declaration of Helsinki and adhered to all relevant guidelines and regulations.

## Results

### Sociodemographics

Of the 119 participants, 79.0% identified as male (*n* = 94), 16.8% as female (*n* = 20), three people as diverse, and two as trans persons. The average age was 42 years, with the youngest participant being 22 years old and the oldest 67. Most participants were unemployed (61.7%) or retired (8.8%). Only 2% earned income through work. 65.5% of respondents were born in Germany, while 34.5% were born in another country. These individuals came from Turkey (6%), Italy (4.2%), Bulgaria, Syria, and Iran (3.4% each), Ukraine (2.5%), Afghanistan, Poland, and Russia (1.7% each), and one person each from Romania, Tunisia, Czechia, Uzbekistan, Greece, Kazakhstan, Lithuania, Congo, and Belgium (see Table 1). Participants were asked where they predominantly slept during the past week. The most common response was sleeping outside (32.2%), followed by sleeping in their own apartment (21.2%). Less commonly, respondents mentioned staying with friends (14.4%), in emergency shelters (10.2%), or in housing projects (9.3%). The share of homeless individuals, people without own apartment, among the respondents was 78.8%. Additionally, it became evident that people who used heroin, cocaine or crack after 10 PM were more likely to sleep outside. Of those who reported consuming heroin late at night, 50% had slept on the street, compared to only 10.5% of those who consumed heroin earlier in the evening (X²=9.007; *p* < 0.01; df = 1). Of those who used cocaine after 10 PM 63.6% had slept on the street compared to 25% who used cocaine earlier (X²=4.030; *p* < 0.5; df = 1) and all who used crack after 10 PM had slept on the street, but only 28.6% who used crack earlier (X²=7.540; *p* < 0.01; df = 1).

### Substance use

In the past 24 h, heroin was most frequently consumed (64.7%), followed by alcohol (56.3%). Fentanyl was consumed only by one respondent in the past 24 h. For other substances see Table 1. Multiple substances were used by 42.9% of the respondents in the last 24 h. Furthermore, 55.1% of respondents (*n* = 65) have experienced an overdose at least once in their lifetime. Of these, 36.5% reported having had one overdose, 20.6% reported two, 9.5% three, 12.7% four, and 20.7% reported five or more overdoses.

**Table 1:** Sociodemographic and substance use

	*n*	Md (Sd)
Age (*n* = 119)	119	42 (9.83)
	***n***	**%**
Gender identity (*n* = 119)		
Male	94	79.0
Female	20	16.8
Divers	3	2.5
Trans*	2	1.7
Country of birth (*n* = 119)		
Germany	78	65.5
Other	41	34.5
Income (*n* = 102)		
Unemployed	63	61.7
Retired	9	8.8
Employed	2	2.0
Sexwork	2	2.0
Other income (*n* = 119)		
Drug dealing	15	12.6
Begging	11	9.2
Support from family	8	6.7
Stealing	6	5.0
Collecting deposit bottles	5	4.2
Selling a homeless newspaper	2	1.7
Burglary	1	0.8
No health insurance (*n* = 118)	26	22.0
Substances used (24 h-prevalence) (*n* = 119)		
Heroin	77	64.7
Alcohol	67	56.3
Benzodiazepines	39	32.8
THC	30	25.2
Cocain (intravenous, nasal)	30	25.2
Pregabalin (Lyrica)	27	22.7
Cocain / Crack (inhaled)	25	21.0
Fentanyl	1	0.8

Heroin consumption behavior in the sample was examined in more detail. 78.0% reported daily heroin use, 11.0% multiple times per week, 8.5% multiple times per month, and 2.4% once a month or less. The predominant mode of heroin consumption was inhalation (68.5%), followed by intravenous use (29.2%) and nasal use (2.2%). On average, heroin was consumed 5.1 times per day. Of those surveyed, 54.5% were in opioid substitution treatment (OST) at the time of the survey. Table 2 compares the substance use of those in substitution treatment with those not in treatment.

**Table 2:** Substance use (24-hour prevalence) of people in OST vs. not in OST

Variable	OST (*n* = 63)		not in OST (*n* = 55)		statistic	
	*n*	%	*n*	%	X²	*p*
Heroin	27	42,9	50	90,9	29,904	< 0.001
Cocaine	13	20,6	16	29,1	1,133	0.287
Crack	7	11,1	18	32,7	8,217	0.004
Alcohol	44	69,8	22	40,0	10,609	0.001
Benzodiazepines	26	41,3	12	21,8	5,089	0.024
THC	22	34,9	8	14,5	6,430	0.011

### Cocaine and crack use

Most respondents (64.5%) reported daily cocaine use. The average number of daily consumption events for intravenous or nasal cocaine use was 3.6 (minimum 1, maximum 12), while the average number of daily crack-smoking events was 5.9 (minimum 1, maximum 30). The high-frequency consumption patterns in crack smoking were only reported by five individuals. Cocaine was predominantly consumed as crack by inhalation (57.1%). A total of 37.4% reported intravenous cocaine use, and 5.5% nasal cocaine consumption.

An open question asked about motives for crack consumption. Curiosity about experiencing a strong high was mentioned as a frequent motive. Social aspects of group consumption also played a role. Some users reported experiencing a powerful (positive) high. Some users described the effect as highly positive, providing a temporary escape from problems. However, users frequently expressed a desire to immediately relive the experience.

### Characteristics of people who use crack (PWUC)

It was found that PWUC were, on average, significantly younger (36.56 years) than people who do not use crack (43.22 years). PWUC had a higher chance of sleeping outdoors in the past week. Moreover, significantly fewer PWUC (28.0%) were in OST than those who reported not using crack (60.2%). Respondents who currently used crack were significantly less likely to have consumed alcohol in the past 24 h. However, there was no significant difference regarding heroin use in the past 24 h (see also Table 3).

**Table 3:** Characteristics of people who use crack

Variable	PWUC		Other PWUD		Statistic	
	M	SD	M	SD	t-Test	*p*
Age (*n* = 119)	36.56	11.25	43.22	8.97	3.122	0.002
	**n**	**%**	**n**	**%**	**X²**	***p***
Sleeping outside	14	56.0	24	25.8	3.602	0.004
Heroin use (last 24 h)	20	80.0	57	60.6	3,242	0.072
OST	7	28.0	56	60.2	8.217	0.004
Alcohol use (last 24 h)	8	32.0	59	62.8	7.598	0.006

### Psychosocial support needs

The study also explored areas where respondents saw a need for support (*n* = 118). Multiple answers were possible, and ten potential areas of support were listed. The most frequently mentioned need was housing assistance (69.5%) followed by help with bureaucratic assistance (60.2%), and health-related support (51.7%). Specific health related support needs such as detox referral, addiction treatment and OST were less often mentioned. Particularly low were the reported needs in the areas of family/social relationships (3.4%) and employment (3.4%) (see Table 4).

**Table 4:** Psychosocial support needs (*n* = 118)

Needs	*N*	%
Housing	82	69.5
Bureaucratic assistance	71	60.2
Health related support	61	51.7
Legal issues	38	32.2
Detox referral	30	25.4
Addiction treatment referral	22	18.6
OST referral	14	11.9
Finances/Debt	10	8.8
Family/Social relationships	4	3.4
Employment	4	3.4

### Health insurance

Access to medical care, including OST, is significantly limited or impossible without health insurance. Among the respondents (*n* = 118), 22.0% reported not having health insurance. Table 5 shows the differences between PWUD with and without health insurance. PWUD without health insurance were significantly more likely to have a migration background and more likely to sleep outside They were also significantly more likely to have consumed heroin and cocaine in the past 24 h. However, no difference was found regarding crack use.

**Table 5:** Differences between PWUD with and without health insurance

Variable	Without health insurance		With health insurance		Statistic	
	*n*	%	*n*	%	X²	*p*
Migration	16	61.5	25	27.2	10.558	0.001
Sleeping outside	13	50.0	25	27.2	4.838	0.028
Heroin use (last 24 h)	24	92.3	53	57.6	10.765	0.001
Cocaine use (last 24 h)	12	46.2	17	18.5	8.376	0.004
Crack use (last 24 h)	9	34.6	16	17.4	3.602	0.058
Use of drug consumption rooms	24	92.3	74	80.4	2.030	0.154
OST	2	7.7	61	66.3	27.984	< 0.001

### Participant recommendations and individual wishes

Participants were asked open questions about their system related recommendations and individual wishes for themselves (*n* = 113). A significant number of respondents called for an expansion of drug consumption rooms in the city and longer opening hours. One participant recommended: “*The consumption rooms should be bigger and have longer opening hours.*” (Interview 73). Furthermore, there were requests for more emergency shelters and daytime rest areas (e.g. relaxation rooms), as well as specific services for women. The entry controls, who decide who is getting a bed for the night in emergency shelters, were also criticized. The precarious housing conditions of many PWUD were also reflected in their personal feedback. 27 participants expressed the hope for stable housing: “*A place to retreat*,* where you can close the door*.” (Interview 5).

A better-coordinated treatment system, including OST for people without health insurance, was also mentioned. One respondent described the situation as follows: “*Substitution doctors*,* general practitioners and psychiatrists should work together*,* especially regarding medications. I don’t want to play them off against each other*,* but I’m afraid of having my medications taken away*,* so I don’t tell them everything*.” (Interview 40). The desire to overcome substance use or addiction was mentioned by 39 people. Besides concrete measures such as starting OST or therapy, distancing from the ODSC was also mentioned. The wish for improved social relationships was expressed by 22 participants, especially the desire for a partnership and to reconnect with their children.

In other statements respondents called for more empathy, acceptance, and openness from the staff of the harm reduction services, with less judgment and discrimination. This was evident in the following statement: “*More acceptance from the people working in harm reduction services. They always say you need insight that you are addicted*,* but you’re not always treated that way.”* (Interview 75). Additionally, some participants called for a societal shift in how addiction is treated, with more tolerance toward PWUD. One respondent stated: “*That society should show more tolerance toward us*.” (Interview 7). There was also criticism of the unequal treatment of different vulnerable groups, reflecting the pressure on how societal resources are allocated.

## Discussion

The aim of the current study was to identify the characteristics of the PWUD of the ODSC in Cologne, Germany, the drugs they use, with a special focus on crack, and their needs and problems. First, we discuss the heroin use, as it was the substance with the highest prevalence. Second, we focus on crack use, because there are reports of an increase since 2018 which had a huge impact on the ODSC in Germany [17, 21]. Thirdly, we discuss the overall health status and the housing situation, because these two topics seem to be the most important to the participants of the study.

We found that the most frequently consumed substance was heroin, with a 24-hour prevalence of 64.5%. This corresponds to approximately 69% of syringes used in the syringe analysis project ESCAPE by the European Drug Agency (EUDA) in Cologne, where heroin residues were detected [26]. 54.4% of participants were in OST, which is slightly more than in Germany overall [23] and should also be increased in Cologne. In Germany, opioids were once again responsible for most deaths related to illicit drugs in 2023 [27]. At the same time, the number of deaths related to illicit drugs in Germany has doubled over the past 10 years [28]. Similarly, an increase in deaths related to illicit drugs has been observed in Cologne, where the number of deaths rose from 41 in 2014 to 97 in 2023 [29]. Deaths related to illicit drugs are often associated with opioid overdoses [30]. In the current study, 55.1% of the respondents had experienced at least one overdose during their lifetime. Opioid overdoses frequently occur at home, often in the presence of others [31]. Take-home naloxone could prevent fatal outcomes in these situations [32] and are not widely implemented yet, neither in Germany nor specifically in Cologne [33]. Additionally, many reported using heroin for the last time after 10 PM. These findings support extending the operating hours of drug consumption rooms to ensure safer consumption opportunities during late hours. While one of the two drug consumption rooms is open until 11 PM, the other one closes at 7:45 PM. Allowing drug use in emergency shelters, which is forbidden now, could also create a safer environment. It could also decrease the risk of sleeping on the streets for people who use substances after 10 PM.

The increase in crack consumption has been reported in several German cities in recent years and is a new challenge for the local drug services [17]. While crack use was previously concentrated in Frankfurt, Hamburg, and Hanover, this pattern is changing [19]. Systematically collected reports of drug services show that crack consumption has also been observed in the ODSC of other cities since 2018 [17, 21]. The 24-hour prevalence of crack in this study was 21%, which is significantly lower than in other German cities as Frankfurt (77%) [34] and Bremen (60–65%) [11]. The lower crack consumption in Cologne could be because PWUD had to prepare the cocaine for smoking themselves. Readily prepared crack, like is the case in Frankfurt, was not available for purchase at the time of the survey. These changes in consumption habits should be addressed by adjusting harm reduction services accordingly. Drug consumption rooms should have designated areas for smoking, with easy access for frequent use [35]. This trend from intravenous consumption to inhalation is already reported for North-Rhine-Westphalia [20] and is also relevant for people who use heroin. In addition, relaxation rooms could provide a space for recovery after binge use, as the Swiss Society of Addiction Medicine recommended in light of the increased use of crack in Switzerland [36]. Relaxation rooms are a space where people can rest and sleep in a safe and quite environment during the day. Binge use of crack was rarely observed in this study and therefore relaxation rooms might not be as relevant as it seemed to be. Still crack is used more often than heroin and therefore following the Zurich model, tolerating micro-dealing between PWUD in harm reduction services could make it easier for people to be there and further reduce risks compared to the ODSC environment [37]. A key element for the treatment of people who use opioids is OST. Since no substitution therapy exists for cocaine, research into substitution-based cocaine treatment should be intensified, and trials of prescription cocaine for users who are dependent should be conducted [36]. This could be especially relevant for people who use crack and are part of the ODSC in Cologne.

The proportion of PWUD without health insurance was 21%. These individuals have access to harm reduction services (e.g. drug consumption rooms, needle and syringe exchange) but they do not have access to OST in Cologne, qualified detoxification treatment, or psychiatric care. During the COVID-19 pandemic, Hamburg piloted low-threshold OST for individuals without health insurance [38]. This type of service should be expanded to cover PWUD in ODSC. PWUD without health insurance were significantly more likely to have a migration background, highlighting a lack of access to above mentioned addiction support services and legal barriers to rehabilitation (e.g. immigration status) [39, 40]. While less participants are in need for detox referral (25,4%), addiction treatment referral (18,6%) and OST referral (11,9%) more than half of the participants (51,7%) report on support needs regarding their general health status. Low threshold medical services, besides addiction treatment, should be available for PWUD, especially for PWUD without health insurance. Bye and colleagues [41] found in a qualitative study that PWUD are also asking for more basic hygienic conditions, what furthermore could, additionally to easy accessible basic health care, improve the environment of an ODSC. The results also illustrate the precarious housing situation among PWUD: only 21.2% had their own apartment, while 56.8% had no permanent housing. This situation is reflected in the high demand for housing support, as 69.5% of respondents expressed a need for assistance in finding accommodation. Open questions about personal wishes further emphasized this housing crisis. Similarly, in Frankfurt, 62% of people of the drug scene were reported as homeless [12]. Homeless individuals face a higher mortality rate [42]. In Cologne, 398 people were estimated to be street homeless, with a total of 11,740 homeless people [43]. Housing First projects could help meet this need in Cologne [44]. Although Cologne offers emergency shelters, of which 10.2% of respondents reported using during the previous week, 32.2% reported sleeping outside.

### Limitations

The current findings should be interpreted in the light of several limitations. First, the survey may not be representative due to the unknown total population size, and the use of non-random sampling [45]. Individuals already engaged in the support system were likely easier to reach for this type of survey and may therefore be overrepresented in the sample. Furthermore, only individuals who could speak German or English were included, which may have resulted in the underrepresentation of individuals with a migration backgrounds. Therefore, the overall health status, support engagement, and health insurance coverage of PWUD may be worse than what is reflected in this sample. Second, some group comparisons were based on small sample sizes, which necessitates cautious interpretation of these findings. Third, the housing variable is probably susceptible to weather conditions. During the survey period, the weather was already relatively warm, which may have resulted in a greater prevalence of outdoor sleeping than would have been during winter. To obtain a more accurate representation of this variable, it is essential to collect data at different times of the year.

## Conclusion

Referring to the risk environment framework by Rhodes [6] regarding ODSC, it can be concluded that more non-repressive measures like drug consumption rooms can improve the environment for PWUD towards a safer environment with fewer health risks. The introduction of another drug consumption room in Cologne has already provided drug users with an additional harm reduction resource. Considering the increasing crack use, harm reduction services and especially drug consumption rooms need to adapt to the specific impacts of crack use. Furthermore, Housing First projects could improve the precarious living conditions of PWUD. Additionally, a low-threshold OST with sufficient capacity is essential for the well-being of PWUD in Cologne’s ODSC at Neumarkt. Access to OST and basic healthcare is especially problematic for people without health insurance. For understanding the needs of PWUD and health risks associated with the change of consumption patterns longitudinal studies are needed.

## Data Availability

The datasets used and analysed during the current study are available from the corresponding author on reasonable request.
